# Data on the application of the molecular vector machine model: A database of protein pentafragments and computer software for predicting and designing secondary protein structures

**DOI:** 10.1016/j.dib.2019.104815

**Published:** 2019-11-19

**Authors:** Vladimir Karasev

**Affiliations:** St. Petersburg State Electrotechnical University, Prof. Popov str. 5, 197376, St. Petersburg, Russia

**Keywords:** Molecular vector machine, Database of protein pentafragments, Software for predicting and design the secondary protein structure

## Abstract

Based on ideas about the molecular vector machine of proteins [1], a database of protein pentafragments has been created and algorithms have been proposed for predicting the secondary structure of proteins according to their primary structure and for designing the primary protein structure for a given secondary structure that it takes on. A comprehensive software suite (Predicto @ Designer) has been developed using the pentafragments database and the said algorithms. For the proteins used to create the pentafragments database, a high accuracy (close to 100%) in predicting the secondary protein structure as well as good prospects for its use for designing secondary structures of proteins have been demonstrated.

**Table:** Specifications Table

Subject	biology
Specific subject area	database of protein pentafragments and computersoftware
Type of data	Table Figure Datebase Software Image (x-ray) Text file
How data were acquired	Computer software Protein_3D, Predicto @ Designer
Data format	Raw and Analysed
Parameters for data collection	The primary structure of the protein
Description of data collection	By using a database and computer programs
Data source location	Source of protein isolation (animal or plant species)
Data accessibility	Data are with this article
Related research article	Vladimir Karasev, BioSystems 180 (2019) 7–18, https://doi.org/10.1016/j.biosystems.2019.02.001

**Table:** 

**Value of the Data** • A database of protein pentafragments, sorted according to a binary description of their structure. • A computer program Predicto @ Designer using this database and algorithm has been written. • This program may be useful in the problems of predicting and designing of protein structure. • The obtained data can contribute to the development of a database and computer software.

## 1 Data

**Fig. 1:**
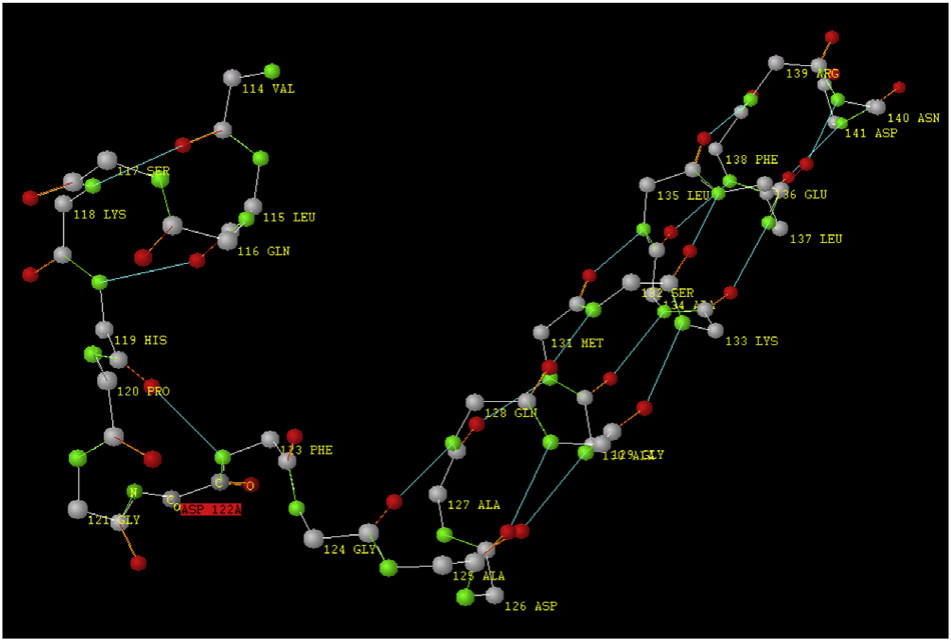
Fragment 114–141 of the polypeptide chain of porcine myoglobin [4].

In this paper, software is described based on the model [1]. The process of predicting secondary protein structure described in the patent [2]. An example of prediction result is given in Table 1, A (a fragment of porcine myoglobin [3]). This fragment illustrates that the whole fragment under consideration can be predicted as a sequence of 10-digit numbers. The comparison with structured experimental data [4], visualized with “Protein 3D” software [5], proved that the software predicts this structure correctly (Fig. 1).

**Table 1:** Predicting secondary myoglobin structure without correction (A) and with correction based on the replacement of amino acids in pentafragments (B).

A. Without correction		B. Correction based on the replacement of amino acids
Pig (Pig without coorection.dbkx)	Alligator (ALLIGAT without coorection.dbkx)	Alligator (ALLIGAT amino acid correction.dbkx)
141 XXX D Asp 1111111111	142 XXX D Asp 1111111111	142 XXX D Asp 1111111111
140 XXX N Asn 1111111111	141 XXX N Asn 1111111111	141 XXX N Asn 1111111111
139 XXX R Arg 1111111111	140 XXX R Arg 1111111111	140 XXX R Arg 1111111111
138 XXX F Phe 1111111111	139 XXX F Phe 1111111111	139 XXX F Phe 1111111111
137 XXX L Leu 1111111111	138 XXX L Leu 1111111121	138 XXX L Leu 1111111111
136 XXX E Glu 1111111111	137 XXX E Glu	137 XXX E Glu 1111111111
135 XXX L Leu 1111111111	136 XXX L Leu	136 XXX L Leu 1111111111
134 XXX A Ala 1111111111	135 XXX A Ala	135 XXX A Ala 1111111111
133 XXX K Lys 1111111111	134 XXX K Lys	134 XXX K Lys 1111111111
**132 XXX S Ser 1111111111**	**133 XXX R Arg**	**133 XXX R *Arg* 1111111111 ASN**
131 XXX M Met 1111111101	132 XXX M Met	132 XXX M Met 1111111101
130 XXX A Ala 1111110101	131 XXX A Ala	131 XXX A Ala 1111110101
**129 XXX G Gly 1111010101**	**130 XXX A Ala**	**130 XXX A *Ala* 1111010101 GLY**
**128 XXX Q Gln 1101010101**	**129 XXX Q Gln**	**129 XXX Q *Gln* 1101010101**
**127 XXX A Ala 0101010110**	**128 XXX S Ser**	**128 XXX S *Ser* 0101010110 ALA**
126 XXX D Asp 0101011000	127 XXX D Asp	127 XXX D Asp 0101011030
125 XXX A Ala 0101100000	126 XXX A Ala	126 XXX A Ala 0101103000
124 XXX G Gly 0110000010	125 XXX G Gly	125 XXX G Gly 0110300000
123 XXX F Phe 1000001011	124 XXX F Phe	124 XXX F Phe 1030000012
122 XXX D Asp 0000101110	123 XXX D Asp	123 XXX D Asp 3000001210
**121 XXX G Gly 0010111010**	**122 XXX A Ala 0200000000**	**122 XXX A *Ala* 0000121010 GLY**
120 XXX P Pro 1011101011	121 XXX P Pro 0000000000	121 XXX P Pro 0012101010
**119 XXX H His 1110101111**	**120 XXX Y Tyr**	**120 XXX Y *Tyr* 1210101011 HIS**
118 XXX K Lys 1010111111	119 XXX K Lys 0000000000	119 XXX K Lys 1010101111 ARG
**117 XXX S Ser 1011111111**	**118 XXX E Glu**	**118 XXX E *Glu* 1010111111 SER**
**116 XXX Q Gln 1111111111**	**117 XXX A Ala 0000000000**	**117 XXX A *Ala* 1011111111 HIS**
**115 XXX L Leu 1111111111**	**116 XXX I Ile**	**116 XXX I *Ile* 1111111111 LEU**
114 XXX V Val 1111111111	115 XXX V Val	115 XXX V Val 1111111111

Bold indicate substitutions of amino acids in the polypeptide chain at which the prediction in column B occurs. The substituted amino acids used are shown in this column to the right.

**Correction of prediction**. Since our approach uses digital description of pentafragment conformations, replacement of a single amino acid has an impact on prediction accuracy, which is a disadvantage of this method. In this situation, if some pentafragment is missing in the database for any reason, a gap in the structure is predicted, which is clearly seen in Table 1, A on the example of alligator's myoglobin fragment [5]. However, this disadvantage can be rectified by employing correction methods that we have developed [6]. A method for replacement of amino acids is the most interesting among them (See below).

The results given by this method are shown on the example of alligator myoglobin, whose primary structure was determined by Ref. [7]. Whereas the results in the middle column in Table 1, to which correction was not different amino acids in i-th position, then it is possible to replace the original pentafragment search with the search for pentafragment with similar structure but with amino acid changed in i-th position.

## 2 Experimental design, materials, and methods

### 2.1 Creating the database of protein pentafragments

Text files describing hydrogen bonds in the secondary structure of proteins were obtained on the basis of about 2333 PDB-files of the Protein Data Bank (subunits – 2446). The list of proteins is given in the appendix. With the help of the *Protein 3D* program developed by us [5] (the program is free to download), these files were processed in a step-by-step fashion using mini-programs with a view to obtaining and sorting pentafragments. The steps are listed below.

#### 2.1.1 Obtaining text files

Open the source PDB file using the *Protein 3D* program. The *Rendering* icon in the *CIHBS* settings submenu will show us the type of protein with a specification of its hydrogen bond systems. Next, in the *CIHBS* icon, check the box against the line item named *Trace in memory*. Open the bond types table from the *Select bond types* line item using the dropdown arrow, check the boxes against the N*i*H … O*i*–3 and N*i*H … O*i*–4 bonds, and uncheck the *Show all* line item. Next, click on the *Show selected bonds* line item and click *OK*. This will open a window with information about the H-bonds of the protein. After clicking the *Save links* button, we will get a text file with a description of these links. Table 2, A shows a sample fragment from a 1MWC text file (*Sus scrofa* myoglobin).

**Table 2:** Individual stages of how pentafragments to be inserted in the database are obtained.

A	B	C	D
Fragment from a text file (1MWD text file.txt)	Fragment from an inverted text file (inv_1MWD inverted text file.txt)	Examples of pentafragments obtained by cutting (rezfile cutting.txt)	Example of simplified file (sim_2111111211.txt)
114 VAL 114 VAL N - 110 ALA O 114 VAL O - 118 LYS N 115 LEU 115 LEU N - 111 ILE O 115 LEU O - 119 HIS N 116 GLN 116 GLN N - 112 ILE O 116 GLN O - 120 PRO N 117 SER 117 SER N - 113 GLN O 118 LYS 118 LYS N - 114 VAL O 119 HIS 119 HIS N - 115 LEU O 119 HIS O - 123 PHE N 120 PRO 120 PRO N - 116 GLN O 121 GLY 122 ASP 123 PHE 123 PHE N - 119 HIS O 124 GLY 124 GLY O - 128 GLN N 125 ALA 125 ALA O - 129 GLY N	125 ALA O - 129 GLY N 125 ALA 124 GLY O - 128 GLN N 124 GLY 123 PHE N - 119 HIS O 123 PHE 122 ASP 121 GLY 120 PRO N - 116 GLN O 120 PRO 119 HIS O - 123 PHE N 119 HIS N - 115 LEU O 119 HIS 118 LYS N - 114 VAL O 118 LYS 117 SER N - 113 GLN O 117 SER 116 GLN O - 120 PRO N 116 GLN N - 112 ILE O 116 GLN 115 LEU O - 119 HIS N 115 LEU N - 111 ILE O 115 LEU 114 VAL O - 118 LYS N 114 VAL N - 110 ALA O 114 VAL	1MWC 120 PRO N - 116 GLN O 120 PRO 119 HIS O - 123 PHE N 119 HIS N - 115 LEU O 119 HIS 118 LYS N - 114 VAL O 118 LYS 117 SER N - 113 GLN O 117 SER 116 GLN O - 120 PRO N 116 GLN N - 112 ILE O 116 GLN 1MWC 119 HIS O - 123 PHE N 119 HIS N - 115 LEU O 119 HIS 118 LYS N - 114 VAL O 118 LYS 117 SER N - 113 GLN O 117 SER 116 GLN O - 120 PRO N 116 GLN N - 112 ILE O 116 GLN 115 LEU O - 119 HIS N 115 LEU N - 111 ILE O 115 LEU 1MWC 118 LYS N - 114 VAL O 118 LYS 117 SER N - 113 GLN O 117 SER 116 GLN O - 120 PRO N 116 GLN N - 112 ILE O 116 GLN 115 LEU O - 119 HIS N 115 LEU N - 111 ILE O 115 LEU 114 VAL O - 118 LYS N 114 VAL N - 110 ALA O 114 VAL	1THB 136 GLY 135 ALA 134 VAL 133 VAL 132 LYS 1HDS 134 ALA 133 VAL 132 VAL 131 LYS 130 GLN 1THB 69 ALA 68 ASN 67 THR 66 LEU 65 ALA 1AZI 67 VAL 66 THR 65 GLY 64 HIS 63 LYS

#### 2.1.2 Inverting text files

For the Predicto @ Designer program to work, the amino acid sequences contained in our pentafragments database need to be written from bottom to top. This pattern simulates the protein synthesis process, which evolves from the N-end to the C-end. The *Invertor* program takes the data written in the text file and rearranges them from the bottom up (Table 2, B).

#### 2.1.3 Cutting text files into pentafragments

Using the *cutter_u* program, cut the inverted files into pentafragments that will store information about the arrangement of H-bonds. Cutting is done by shifting the frame by one amino acid. Table 2, C shows some examples of such pentafragments.

#### 2.1.4 Sorting and simplifying pentafragments

Use the *Selector* program to sort the pentafragments obtained as shown above in accordance with the link encoding system we have adopted (see Table 3, Table 4). Use the *Simplification* program to simplify the files obtained (Table 2, D).

**Table 3:** Notations of bonds in text PDB-files (A), types of H-bonds (B), their coding with Boolean pairs of variables (C). an example of pentafragment (D) and its 10-digit description (E).

А. Notations in text PDB-files	B. Types of H-bonds	C. Coding	D. An example of pentafragment and its coding	
X_1_X_2_ Abc	**No H-bonds** No H-bonds	00	**51 Gln O - 55 Glu N** **51 Gln** 50 Pro 49 Ala 48 Asp 47 Ser	**01** 00 00 00 00
X_1_X_2_ Abc O–Y_1_Y_2_ Deh N X_1_X_2_ Abc	**Н-bond only with C**=**O-group**	01		
X_1_X_2_ Abc N–Y_3_Y_4_ Ehf O X_1_X_2_ Abc	**H-bond only with NH-group**	10		
X_1_X_2_ Abc O–Y_1_Y_2_ Deh N X_1_X_2_ Abc N–Y_3_Y_4_ Ehf O X_1_X_2_ Abc	**H-bonds both with C**=**O and with NH-group**	11	**E. 10-digit descriptions of PFs and file names**	
			**01**00000000	

In cell D, the selected first two lines correspond to the highlighted designation 01 in cell E.

**Table 4:** Coding of types of H-Bonds in the form of binary combinations for an improved database of pentafragments.

№	Types of H-bonds	Binary Combinations							
		Bonds	Code	Bonds	Code	Bonds	Code	Bonds	Code
α-helix									
1.	N_i_H … O_i-4_ O_i-4_ … HN_i_	0 0	00	0 1	01	1 0	10	1 1	11
Inverted α-helix									
2.	N_i_H … О_i+4_ О_i_ … HN_i-4_	0 0	00	1 0	70	0 1	07	1 1	77
helix 3_10_									
3.	N_i_H … O_i-3_ O_i-3_ … HN_i_	0 0	00	0 1	03	1 0	30	1 1	33
Inverted helix 3_10_									
4.	N_i_H … О_i+3_ О_i_ … HN_i-3_	0 0	00	1 0	60	0 1	06	1 1	66
Combination of α-helix and helix 3_10_									
5.	N_i_H … O_i-4_ … O_i-3_ O_i-4_…HN_i_⋯HN_i-1_	0 0	00	0 2	02	2 0	20	2 2	22
Combination of Inverted α-helix and helix 3_10_									
6.	N_i_H … O_i+4_ … O_i+3_ O_i_ … HN_i-4_ … HN_i-3_	0 0	00	2 0	40	0 2	04	2 2	44

An identification system was developed to sort pentafragments in database folders based on the binary coding of H-bonds [[8], [9], [10], [11]]. An example of describing the structure of pentafragments with the help of implemented coding is given in Table 3. In this case, the 10-digit numbers describing a conformation of pentafragments were transferred to the file names (Table 3, E).

Subsequently, this coding procedure became more complicated (Table 4). Additional figures to identify various types of secondary structures were introduced, but retained its binary principle [11].

The structure of the database organized in accordance with the link encoding system as per Table 4 is shown in Table 5. It consists of folders containing pentafragment files and designated by the i^th^ pair of variables (see the *Folder numbering* column, Table 5), of files enclosed in these folders and containing 10-digit numbers that describe the structure of the pentafragments (column 2), and of pentafragments contained in these files and associated to their specific positions in proteins (column 3). To speed up the search for pentafragments, the software has the database written in the form of strings (see Ref. [6] for an example).

**Table 5:** Pentafragment database structure.

Folder numbering (Database.JPG)				Pentafragment files. **Folder 37-X**X (Pentafragment Files of Folder 37-00.JPG)	Pentafragments of the file 37**30000373.txt** (Pentafragment of File 3730000373.JPG)
No.	Folder	No.	Folder		
1 2 3 4 5 6 7 8 9 10 11 12 13 14 15 16 17 18 19	00-XX 01-XX 02-XX 03-XX 04-XX 06-XX 07-XX 10-XX 11-XX 12-XX 13-XX 14-XX 16-XX 17-XX 20-XX 21-XX 22-XX 23-XX 27-XX	20 21 22 23 24 25 **26** 27 28 29 30 31 32 33 34 35 36 37 38	30-XX 31-XX 32-XX 33-XX 34-XX 36-XX **37-XX** 40-XX 43-XX 60-XX 61-XX 62-XX 63-XX 66-XX 70-XX 71-XX 72-XX 74-XX 77-XX	3700000270.txt 3700000370.txt 3700003270.txt 3700003370.txt 3700037270.txt 3703000370.txt **3730000373.txt** 3730003373.txt	DKK 23 TYR 22 GLY 21 ARG 20 TYR 19 ASN 2BQA 23 ILE 22 GLY 21 ARG 20 TYR 19 GLY 2JIZ 294 TYR 293 ALA 292 GLU 291 ARG 290 GLY 3D27 65 TYR 64 GLY 63 HIS 62 TYR 61 GLY

### 2.2 Program layout

The computer program named PREDICTO @ DESIGNER The program is written in C ++. It has been registered [12] as well as described in detail in Ref. [13]. For the program, a file of the.pdb format (Protein Data Bank) and.gen (Genbank) can be used, which are transformed by the program into the.dbk format (Table 6, A) in which the program predicts the secondary structure of the protein. The result of the program is written in.dbkx format (Table 6, B).

**Table 6:** Formats used by the program PREDICTO @ DESIGNER.

A					B					
A fragment of the pig myoglobin protein (1MWC file) in.dbk format (1MWD_A.dbk)					Recording the result of the program in.dbkx format (1MWD_A.dbkx)					
15	XXX	G	GLY	bbbbbbbbbb	15	XXX	G	GLY	1112121011	3K9Z 1DMR
14	XXX	W	TRP	bbbbbbbbbb	14	XXX	W	TRP	1212101111	3K9Z 1DMR
13	XXX	V	VAL	bbbbbbbbbb	13	XXX	V	VAL	1210111111	3K9Z 1MWC
12	XXX	N	ASN	bbbbbbbbbb	12	XXX	N	ASN	1011111111	3K9Z 1MWC
11	XXX	L	LEU	bbbbbbbbbb	11	XXX	L	LEU	1111111111	3K9Z 1MWC
10	XXX	V	VAL	bbbbbbbbbb	10	XXX	V	VAL	1111111101	3K9Z 1MWC
9	XXX	L	LEU	bbbbbbbbbb	9	XXX	L	LEU	1111110101	3K9Z 1MWC
8	XXX	Q	GLN	bbbbbbbbbb	8	XXX	Q	GLN	1111010101	3K9Z 1DMR
7	XXX	W	TRP	bbbbbbbbbb	7	XXX	W	TRP	1101010101	3K9Z 1DMR
6	XXX	E	GLU	bbbbbbbbbb	6	XXX	E	GLU	0101010100	3K9Z 1DMR
5	XXX	G	GLY	bbbbbbbbbb	5	XXX	G	GLY	0101010000	3K9Z 1DMR
4	XXX	D	ASP	bbbbbbbbbb	4	XXX	D	ASP	bbbbbbbbbb	
3	XXX	S	SER	bbbbbbbbbb	3	XXX	S	SER	bbbbbbbbbb	
2	XXX	L	LEU	bbbbbbbbbb	2	XXX	L	LEU	bbbbbbbbbb	
1	XXX	G	GLY	bbbbbbbbbb	1	XXX	G	GLY	bbbbbbbbbb	
0	ATG	M	MET	bbbbbbbbbb	0	ATG	M	MET	bbbbbbbbbb	

**Fig. 2:**
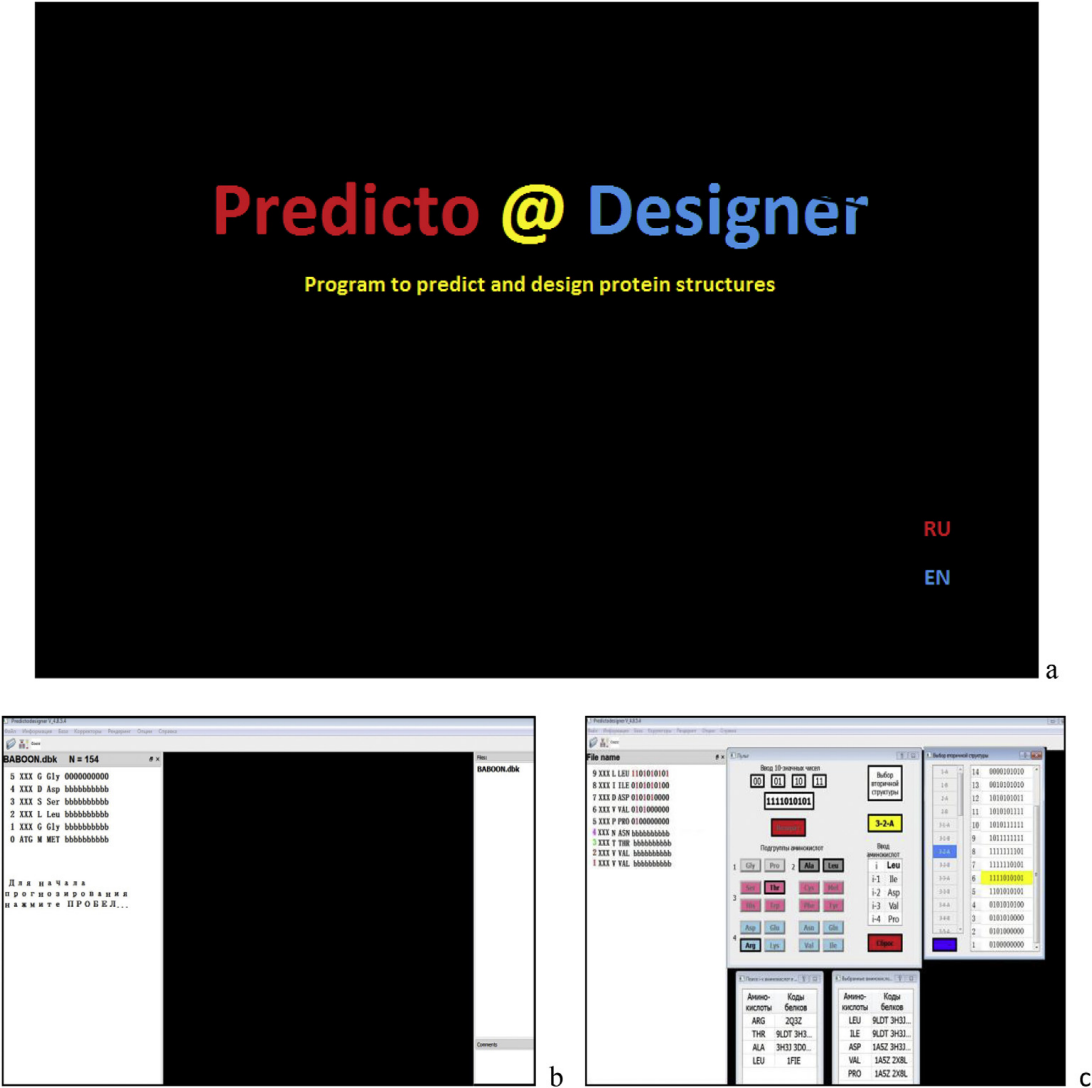
The startup screen and workspaces of the PREDICTO @ DESIGNER program. a – program startup screen; b – PREDICTO section workspace; c – DESIGNER section workspace.

Fig. 2, a shows the startup screen of the PREDICTO @ DESIGNER program. Clicking on the word PREDICTO sets the program to the secondary protein structure prediction mode (Fig. 2, b shows the workspace where digital and structural information is displayed) and clicking on the word DESIGNER sets it to the design mode (Fig. 2, c shows the workspace, control panel, and icons used to display information required for the design).

### 2.3 The procedure for prediction

The method of predicting secondary protein structure described in the patent [2] consists in isolating pentafragments in a file with specially formatted primary structure of proteins (files.dbk) and their search in the Database. Since every pentafragment has a 10-digit identification number in the Database, the software reads the code number of the found pentafragment and displays it onto the numeric operating field in a bottom-up sequence progressively as pentafragments are selected in a protein chain from start to finish. This procedure consists of two stages: an initial pentafragment is found at the first stage and if it is detected correctly then the remaining protein is predicted further at the second stage [2]. It has been found that when applying this approach, the secondary structure of all proteins used to develop the database is predicted with an accuracy close to 100%.

### 2.4 Prediction correction method by replacement of amino acids

The method consists in the following [6]. Let us assume that at some i-th stage the software has isolated a pentafragment to be searched for that has not been found under a code number defined on the basis of search algorithm. If this pentafragment could be found at the previous i-1-th stage, then it is all about the amino acid that appeared in the pentafragment at the i-th stage. It is well known that these changes (mutations) are frequently observed for the same type proteins but extracted from different kinds of organisms. Because the search for pentafragment with missing i-th amino acid should be conducted under the same folder number, as for the other pentafragments with similar structure but with applied, show quite low prediction accuracy, a region with amino acids from 115 to 138 (Table 1) was completely predictable as a result of applying this method. Comparison of the predicted structure of alligator myoglobin with porcine myoglobin (Table 1, left column) shows that in general both structures have similar position of α-helixes in this fragment. Thus, applying this correction method significantly improves prediction accuracy for secondary structure of proteins.

### 2.5 Further ways to develop the prediction method

Applying the described prediction correction method is convenient and relevant to use for the groups of proteins with similar structure but derived from different species (as in cases with myoglobins and other heme-containing proteins). Ideally, it would be better to have a universal database that could be used to predict secondary structure of any protein with high accuracy. We have shown a practical possibility for creating it [14]. However, a high increase in the number of pentafragments in the database significantly increases the number of alternative options for prediction of secondary structures. This, in its turn, sharply slows down software performance and deteriorates the prediction quality.

Due to the above-mentioned reasons, we believe it is more relevant to develop ad-hoc databases aimed at predicting structurally close proteins. In this case, a universal database can be built on the basis of hierarchical structure of specialized databases. A prediction algorithm will consist of two stages: a) preliminary search of common elements being attributable to certain protein groups; b) final prediction based on a specialized database. There is a lot of work to be done in this respect, but the results of this work seem to be quite promising.

### 2.6 Developing a design method for secondary structures

Because the proposed approach can predict secondary structures of proteins quite accurately, it would be logical to apply the same approach to design secondary structures based on the predefined secondary structure. This method is detailed in the patent of [15]. It is implemented in the Designer section [13] of the Predicto @ Designer software. The initial protein pentafragment and its description in the form of 10-digit number in the binary numeral system is set using the control panel. The selected pentafragment is searched for in the database and, if it is found, then it is necessary to see one new amino acid and 10-digit description of a new pentafragment containing the previous four amino acids and one new and run a new search in the database. If the new pentafragment is found, then the procedure should be repeated.

The description presented in the patent is based on the data available in literature, and therefore, it confirms the feasibility of this design. However, before this method is recommended for a large-scale implementation, it must pass a more comprehensive experimental validation on the basis of up-to-date scientific and engineering know-hows. The studies are being carried out in this respect.

## References

[1] Karasev V.A. (2019). A model of molecular vector machine of proteins. BioSystems.

[2] V.A. Karasev, V.V. Luchinin, A Method of Predicting Secondary Structure of Protein, R F Patent No.2425837 date of publ. 10.08.2011, Bull. No.22 (In Russian).

[3] Akaboshi E. (1985). Cloning and sequence analysis of porcine myoglobin cDNA. Gene.

[4] Krzywda S., Murshudov G.N., Brzozowski A.M., Jaskolski M., Scott E.E., Klizas S.A., Gibson Q.H., Olson J.S., Wilkinson A.J. (1998). Stabilizing bound O_2_ in myoglobin by valine68 (e11) to asparagine substitution. Biochemistry.

[5] Demchenko E.L., Karasev V.A. (2017). http://protein-3d.ru/.

[6] Karasev V.A., Kalinin S.B. (2016). PREDICTO @ DESIGNER computer software for prediction and design of protein secondary structures: UPGRADE. III. Algorithms for searching pentafragments in databases and correction methods for predicting secondary structures of proteins. Biotechnosfera.

[7] Dene H., Sazy J., Goodman M., Romero-Herrera A.E. (1980). The amino acid sequence of alligator (Alligator mississippiensis) myoglobin. Phylogenetic implications. Biochim. Biophys. Acta.

[8] Karasev V.A. (2014).

[9] Karasev V.A., Stefanov V.E. (2013). 10-digits boolean system in description of protein pentafragments. Symmetry: Sci. Cult..

[10] V.A. Karasev, A.I. Belyaev, V.V. Luchinin, Database of Protein Pentafragments, Registered in ROSPAPENT No. 2010620364, 2010. (In Russian).

[11] Karasev V.A., Kalinin S.B. (2016). PREDICTO @ DESIGNER computer software for prediction and design of protein secondary structures: UPGRADE. I. Database of protein pentafragments considering N_i_H….O_i-3_, N_i_H….O_i-4,_ and other types of H-bonds in secondary structures of proteins. Biotechnosfera.

[12] S.B. Kalinin, V.A. Karasev, V.V. Luchinin, Software to Predict Secondary Protein Structure and Design Primary Protein Structure with Defined Secondary Structure (Predicto@Designer). Registered in ROSPATENT, No.2015622295, dated 17.02.2015. (In Russian).

[13] Karasev V.A., Kalinin S.B. (2016). PREDICTO @ DESIGNER computer software for prediction and design of protein structures: theory. Design. Application. Biotechnosfera.

[14] Karasev V.A., Kalinin S.B. (2016). PREDICTO @ DESIGNER computer software for prediction and design of protein secondary structures: UPGRADE. II. Principles of developing theoretical database of protein pentafragments. Biotechnosfera.

[15] V.A. Karasev, V.V. Luchinin, A Method of Designing Primary Structure of Protein with Specified Secondary Structure, RF Patent No.2511002, date of publ. 10.04.2014, Bull. No.10 (In Russian).

