# Supplementary material for: Data on the application of the molecular vector machine model: A database of protein pentafragments and computer software for predicting and designing secondary protein structures
Source: Data Brief. 2019 Nov 19;28:104815. doi: 10.1016/j.dib.2019.104815 (PMC6911939; doi:10.1016/j.dib.2019.104815)
Supplement: Multimedia component 1 [file mmc1.docx]

List of pdb-files

Total processed proteins – 2333

Total processed subunits – 2446

10GS.pdb 1VSD.PDB 2Z01.pdb 3G84.pdb

12AS.pdb 1WHO.PDB 2Z02.pdb 3G85.pdb

19GS.pdb 1WSY.PDB 2Z07.pdb 3G8K.pdb

1a22-B.pdb 1XDN.pdb 2Z08.pdb 3GA5.pdb

1a22.pdb 1XDP.pdb 2Z0K.pdb 3GAP.PDB

1A44.pdb 1XER.PDB 2Z0X.pdb 3GBB.pdb

1A4I.PDB 1XEX-a.pdb 2Z11.pdb 3GBG.pdb

1A4P.PDB 1XEX-b.pdb 2Z22.pdb 3GBV.pdb

1A6Y.PDB 1XEX.pdb 2Z23.pdb 3GCD.pdb

1A94-a.pdb 1xfw-a.PDB 2Z30.pdb 3GCE.pdb

1A94.pdb 1xfw-p.pdb 2Z3V.pdb 3GCG.pdb

1AA2.PDB 1xfw.pdb 2Z43.pdb 3GDE.pdb

1AAY.PDB 1XJO.PDB 2Z4P.pdb 3GDG.pdb

1ABA.PDB 1XKF.pdb 2Z4Q.pdb 3GDW.pdb

1AER.PDB 1XKV.pdb 2Z54.pdb 3GEK.pdb

1AF0.pdb 1XNG.pdb 2Z58.pdb 3GEU.pdb

1AFR.PDB 1XO0.pdb 2Z5P.pdb 3GEY.pdb

1AFR_A.PDB 1XVA.PDB 2Z68.pdb 3GF2.pdb

1AFR_B.PDB 1xwd-a.PDB 2Z69.pdb 3GF6.pdb

1AGD-b.pdb 1xwd-a.txt 2Z6T.pdb 3GFH.pdb

1AGD.PDB 1xwd-b.pdb 2Z6W.pdb 3GFI.pdb

1AGQ.PDB 1xwd-b.txt 2Z6Y.pdb 3GFK.pdb

1AH7.PDB 1xwd-c.pdb 2Z72.pdb 3GFM.pdb

1AII.PDB 1xwd-c.txt 2Z7B.pdb 3GG1.pdb

1AKY.PDB 1xwd.pdb 2Z7E.pdb 3GGH.pdb

1ALD.PDB 1XYF.PDB 2Z8F.pdb 3GGU.pdb

1AMF.PDB 1YAG-a.pdb 2Z8L.pdb 3GH6.pdb

1AMK.PDB 1YAG-g.pdb 2Z8U.pdb 3GH7.pdb

1AMM.PDB 1YAG.pdb 2Z8X.pdb 3GHA.pdb

1AMP.PDB 1YCC.PDB 2Z8Z.pdb 3GI6.pdb

1AMW.PDB 1YFR.pdb 2Z99.pdb 3GI7.pdb

1ANN.PDB 1YTB.PDB 2Z9X.pdb 3GIX.pdb

1AOK.PDB 1YUN.pdb 2ZA6.pdb 3GJB.pdb

1AOZ.PDB 1ZAO.pdb 2ZAU.pdb 3GJW.pdb

1APX.PDB 1ZSR.pdb 2ZB9.pdb 3GJZ.pdb

1APY-b.pdb 1ZTW.pdb 2ZBI.pdb 3GK6.pdb

1APY.PDB 1ZXQ.PDB 2ZBV.pdb 3GKN.pdb

1AQ2.pdb 256B.PDB 2ZBZ.pdb 3GKT.pdb

1ARB.PDB 2A42.pdb 2ZC2.pdb 3GKV.pdb

1ASH.PDB 2A84.pdb 2ZCI.pdb 3GKX.pdb

1ASO.PDB 2ADM.PDB 2ZCN.pdb 3GL3.pdb

1AT9.PDB 2AE2.PDB 2ZCW.pdb 3GL5.pdb

1ATN-a.pdb 2aew.pdb 2ZCX.pdb 3GLN.pdb

1ATN-d.pdb 2AFG.PDB 2ZDB.pdb 3GLX.pdb

1ATN.PDB 2AG2.pdb 2ZE7.pdb 3GMF.pdb

1AU1.pdb 2AK3.PDB 2ZF9.pdb 3GMG.pdb

1AU7.PDB 2AOG.pdb 2ZGZ.pdb 3GMI.pdb

1AVH.PDB 2APR.PDB 2ZIB.pdb 3GMT.pdb

1AWC-a.pdb 2ARU.pdb 2ZJ4.pdb 3GN5.pdb

1AWC-b.pdb 2ASP.pdb 2ZJ7.pdb 3GN9.pdb

1AWC.PDB 2ATW.pdb 2ZJ8.pdb 3GNJ.pdb

1AYL.pdb 2AYH.PDB 2ZKZ.pdb 3GNW.pdb

1AZI.pdb 2B3J.pdb 2ZL8.pdb 3GOQ.pdb

1B0U.pdb 2B3Y.pdb 2ZNM.pdb 3GP6.pdb

1B6L.pdb 2BBS.pdb 2ZOD.pdb 3GQ0.pdb

1B72.PDB 2BIY.pdb 2ZOU.pdb 3GQ1.pdb

1B76.pdb 2BOP.PDB 2ZOV.pdb 3GQH.pdb

1B7A.pdb 2BQA.pdb 2ZOZ.pdb 3GQJ.pdb

1B8A.pdb 2BU2.pdb 2ZPM.pdb 3GQM.pdb

1B8I-a.pdb 2BUE.pdb 2ZQ0.pdb 3GR0.pdb

1B8I-b.pdb 2BUP.pdb 2ZRQ.pdb 3GR5.pdb

1B8I.PDB 2C0D.pdb 2ZTG.pdb 3GRG.pdb

1B8V.PDB 2C2C.PDB 2ZVC.pdb 3GRS.PDB

1BAG.PDB 2C6U.pdb 2ZVD.pdb 3GRY.pdb

1BAS.PDB 2C8J.pdb 2ZVR.pdb 3GS7.pdb

1BBH.PDB 2C96.pdb 2ZVU.pdb 3GS9.pdb

1BCC-a.pdb 2CBP.PDB 2ZVY.pdb 3GSZ.pdb

1BCC-b.pdb 2CCY.PDB 2ZWJ.pdb 3GUS.pdb

1BCC-c.pdb 2CE0.pdb 2ZWO.pdb 3GUV.pdb

1BCC-d.pdb 2CE9.pdb 2ZY1.pdb 3GV0.pdb

1BCC-e.pdb 2CGP.PDB 2ZYC.pdb 3GV1.pdb

1BCC-f.pdb 2CLU.pdb 2ZYL.pdb 3GVE.pdb

1BCC-g.pdb 2CMT.pdb 2ZYO.pdb 3GVL.pdb

1BCC-h.pdb 2CNA.PDB 2ZZ8.pdb 3GW2.pdb

1BCC-j.pdb 2CO5.pdb 2ZZX.pdb 3GW3.pdb

1BCC.PDB 2CPP.PDB 351C.pdb 3GWQ.pdb

1BCC_2.PDB 2CRK.PDB 3A06.pdb 3GWU.pdb

1BCO.PDB 2CRO.PDB 3A14.pdb 3GX0.pdb

1BCP-a.pdb 2CTS.PDB 3A1I.pdb 3GX1.pdb

1BCP-b.pdb 2CYP.PDB 3A1K.pdb 3GX8.pdb

1BCP-d.pdb 2DGC.PDB 3A1N.pdb 3GY9.pdb

1BCP-f.pdb 2DJH.pdb 3A24.pdb 3GYB.pdb

1BCP-h.pdb 2DOR.PDB 3A2B.pdb 3GYE.pdb

1BCP.PDB 2DY1.pdb 3A2G.pdb 3GYK.pdb

1BCR-a.pdb 2DY3.pdb 3A2Q.pdb 3GYY.pdb

1BCR-b.pdb 2E00.pdb 3A32.pdb 3GYZ.pdb

1BCR.PDB 2E0A.pdb 3A3G.pdb 3GZ1.pdb

1BCX.PDB 2E0Q.pdb 3A3H.pdb 3GZ3.pdb

1BEO.PDB 2E4Q.pdb 3A3P.pdb 3GZ5.pdb

1BES.PDB 2E7P.pdb 3A3U.pdb 3GZG.pdb

1BG1.PDB 2EAV.pdb 3A3X.pdb 3GZH.pdb

1BHM.PDB 2EAX.pdb 3A4F.pdb 3GZI.pdb

1BHT.pdb 2EB1.pdb 3A4J.pdb 3H05.pdb

1BMD.PDB 2EB7.pdb 3A5G.pdb 3H08.pdb

1BNC.PDB 2EBN.PDB 3A6Z.pdb 3H0N.pdb

1BP2.PDB 2ECK.PDB 3A7K.pdb 3H0O.pdb

1BTF.PDB 2EEN.pdb 3A7R.pdb 3H1N.pdb

1BW9.PDB 2EEY.pdb 3A9M.pdb 3H20.pdb

1BWO.pdb 2EF7.pdb 3A9Q.pdb 3H2F.pdb

1BYB.PDB 2EFF.pdb 3AAR.pdb 3H2K.pdb

1C0G.pdb 2EFN.pdb 3AB9.pdb 3H2L.pdb

1C1Y-a.pdb 2EGJ.pdb 3ADK.PDB 3H2M.pdb

1C1Y-b.pdb 2EGR.pdb 3AFF.pdb 3H3G.pdb

1C1Y.PDB 2EHL.pdb 3APP.pdb 3H3J.pdb

1C2R.PDB 2EI4.pdb 3B2M.pdb 3H3Z.pdb

1C3W.PDB 2EIS.pdb 3B2Y.pdb 3H58.pdb

1C7S.PDB 2EJA.pdb 3B39.pdb 3H5J.pdb

1CAY.PDB 2EJB.pdb 3B49.pdb 3H5L.pdb

1CCZ.PDB 2EK4.pdb 3B50.pdb 3H5O.pdb

1CDL.pdb 2EK5.pdb 3B69.pdb 3H5U.pdb

1CDM.pdb 2EPF.pdb 3B6C.pdb 3H5V.pdb

1CDO.PDB 2EPO.pdb 3B6R-a.pdb 3H5Y.pdb

1CDW.PDB 2F02.pdb 3B6R.pdb 3H69.pdb

1CFR.PDB 2F06.pdb 3B6W.pdb 3H6P.pdb

1CFY.PDB 2F2L.pdb 3B79.pdb 3H6W.pdb

1CGO.PDB 2f3y.pdb 3B7E.pdb 3H73.pdb

1cgp.pdb 2F49.pdb 3B7I.pdb 3H75.pdb

1CHK.PDB 2FDN.pdb 3B7L.pdb 3H79.pdb

1CKQ.PDB 2FHA.PDB 3B7P.pdb 3H7G.pdb

1CLC.PDB 2fqm-a.pdb 3B80.pdb 3H8Q.pdb

1CMB.PDB 2fqm.pdb 3B81.pdb 3H8T.pdb

1CNR.PDB 2FVR.pdb 3B8B.pdb 3H93.pdb

1CNT.PDB 2FX2.PDB 3B97.pdb 3H98.pdb

1COL-1.pdb 2FXB.PDB 3B9G.pdb 3H9J.pdb

1COL.PDB 2FXU.pdb 3B9X.pdb 3HA1.pdb

1COX.PDB 2GL6-a.pdb 3BAA.pdb 3HA9.pdb

1COZ.PDB 2GL6.pdb 3BB0.pdb 3HBA.pdb

1CPC.PDB 2GLI.PDB 3BB6.pdb 3HCW.pdb

1CPI.pdb 2GNK.pdb 3BB7.pdb 3HCZ.pdb

1CPQ.PDB 2GST.PDB 3BBL.pdb 3HD5.pdb

1CQX.pdb 2GUF.pdb 3BBR.pdb 3HDC.pdb

1CRK.pdb 2HF7.pdb 3BBY.pdb 3HDD.PDB

1CSN.pdb 2HFT.PDB 3BC7.pdb 3HDF.pdb

1CTF.PDB 2HI2.pdb 3BD2.pdb 3HE0.pdb

1CTN.PDB 2HNK.pdb 3BDA.pdb 3HEF.pdb

1CWM.pdb 2HYK.pdb 3BDD.pdb 3HEP.pdb

1cx4.pdb 2I06.pdb 3BDF.pdb 3HFI.pdb

1D2I.pdb 2IA4.pdb 3BE3.pdb 3HFJ.pdb

1D4X-a.pdb 2ID6.pdb 3BEC.pdb 3HFN.pdb

1D4X-d.pdb 2IDM.pdb 3BED.pdb 3HGL.pdb

1D4X.pdb 2IEK.pdb 3BEN.pdb 3HGM.pdb

1D8W.PDB 2IGL.pdb 3BEO.pdb 3HH1.pdb

1D9Z.pdb 2III.pdb 3BEQ.pdb 3HH8.pdb

1DAK.PDB 2ILK.PDB 3BF4.pdb 3HHD.pdb

1DAP.PDB 2IMK.pdb 3BFP.pdb 3HHF.pdb

1DDG.PDB 2INX.pdb 3BFT.pdb 3hhr.pdb

1DDJ.PDB 2IP6.pdb 3BFU.pdb 3HHV.pdb

1DDT.PDB 2IQ6.pdb 3BGL.pdb 3HHX.pdb

1DED.PDB 2IQY.pdb 3BGY.pdb 3HI2.pdb

1DEJ.pdb 2IRP.pdb 3BH7.pdb 3HIJ.pdb

1DEK.PDB 2IVY.pdb 3BHD.pdb 3HIM.pdb

1DFN.PDB 2IXT.pdb 3BHP.pdb 3HJ0.pdb

1DHP.PDB 2J4X.pdb 3BHQ.pdb 3HJ7.pdb

1DIK.PDB 2J5Y.pdb 3BIH.pdb 3HJH.pdb

1DIM.PDB 2J73.pdb 3BJ5.pdb 3HJO.pdb

1DKK.PDB 2J9G.pdb 3BJ6.pdb 3HKR.pdb

1DLD.PDB 2JBV.pdb 3BJ8.pdb 3HKY.pdb

1DMR.PDB 2JDL.pdb 3BJA.pdb 3HLR.pdb

1DOF.PDB 2JEM.pdb 3BJN.pdb 3HLX.pdb

1DOG.PDB 2JEW.pdb 3BK5.pdb 3HMC.pdb

1DOK.PDB 2JEW.txt 3BKL.pdb 3HNH.pdb

1DPG.PDB 2JG2.pdb 3BKP.pdb 3HNL.pdb

1DQ3.PDB 2JHE.pdb 3BL6.pdb 3HO7.pdb

1DSY.pdb 2JJ7.pdb 3BLJ.pdb 3HOA.pdb

1DT6.pdb 2JJB.pdb 3BLM.PDB 3HOE.pdb

1DTL.pdb 2JJR.pdb 3BNI.pdb 3HOK.pdb

1DV8.PDB 2JJZ.pdb 3BO7.pdb 3HOL.pdb

1DVF.PDB 2JK3.pdb 3BOM.pdb 3HP9.pdb

1DVI.PDB 2JKB.pdb 3BP3.pdb 3HPE.pdb

1DVK.PDB 2JKE.pdb 3BPP.pdb 3HPI.pdb

1DY3.pdb 2JKU.pdb 3BQ6.pdb 3HPW.pdb

1DYT.PDB 2JL4.pdb 3BQG.pdb 3HQ6.pdb

1DZB.PDB 2JLL.pdb 3BQY.pdb 3HQ9.pdb

1DZO.PDB 2LBP.PDB 3BRO.pdb 3HQX.pdb

1E18.PDB 2LHB.PDB 3BRQ.pdb 3HR6.pdb

1E19.PDB 2MBN.PDB 3BRS.pdb 3HR8.pdb

1E2Q.pdb 2MHR.PDB 3BRU.pdb 3HR9.pdb

1E4A.PDB 2MPR.PDB 3BS1.pdb 3HS3.pdb

1E6Y.PDB 2NAD.PDB 3BSO.pdb 3HST.pdb

1E6Y_A.PDB 2NNB.pdb 3BT0.pdb 3HSY.pdb

1E6Y_B.PDB 2NPX.PDB 3BT5.pdb 3HUP.pdb

1E6Y_C.PDB 2nrl.pdb 3BTN.pdb 3HUT.pdb

1E78.pdb 2NTO.pdb 3BTO.PDB 3HUU.pdb

1E79-a.pdb 2NXV.pdb 3BTP.pdb 3HUV.pdb

1E79-d.pdb 2NZ7.pdb 3BV4.pdb 3HV1.pdb

1E79-g.pdb 2O4Q.pdb 3BVP.pdb 3HVA.pdb

1E79-h.pdb 2O5U.pdb 3BVS.pdb 3HVO.pdb

1E79-i.pdb 2O9T.pdb 3BWG.pdb 3HVX.pdb

1E79.pdb 2OBI.pdb 3BWW.pdb 3HXS.pdb

1E7F.PDB 2OBO.pdb 3BWZ.pdb 3HY0.pdb

1E7Q.PDB 2OCX.pdb 3BX1.pdb 3HY2.pdb

1E80.pdb 2ODL.pdb 3BXH.pdb 3HYI.pdb

1EB0.pdb 2OE3.pdb 3BXY.pdb 3HZ4.pdb

1ECA.PDB 2OJP.pdb 3BYL.pdb 3HZ7.pdb

1ECL.PDB 2OK3.pdb 3BYQ.pdb 3HZ8.pdb

1EEH.PDB 2OLP.pdb 3BZK.pdb 3HZS.pdb

1EG4.pdb 2OMF.PDB 3BZN.pdb 3I0X.pdb

1EJB.PDB 2OMX.pdb 3BZO.pdb 3I0Z.pdb

1EJB_1.PDB 2OMZ.pdb 3C04.pdb 3I18.pdb

1EJD.pdb 2OPT.pdb 3C0K.pdb 3I1A.pdb

1EKX.PDB 2OQH.pdb 3C0P.pdb 3I25.pdb

1EMD.PDB 2OQZ.pdb 3C17.pdb 3I3G.pdb

1EMJ.PDB 2OSE.pdb 3C1D.pdb 3I3L.pdb

1EMV.PDB 2OUK.pdb 3C1M.pdb 3I3V.pdb

1emy.pdb 2OVI.pdb 3C1Q.pdb 3I3W.pdb

1ENO.PDB 2OWS.pdb 3C1R.pdb 3I45.pdb

1EPF.pdb 2OWU.pdb 3C1Y.pdb 3I4P.pdb

1EPX.PDB 2OX1.pdb 3C22.pdb 3I4Z.pdb

1EQ6.PDB 2OXL.pdb 3C26.pdb 3I51.pdb

1ERV.PDB 2OZE.pdb 3C2B.pdb 3I57.pdb

1ESF.PDB 2P1X.pdb 3C2U.pdb 3I59.pdb

1ESG.pdb 2P38.pdb 3C2X.pdb 3I5K.pdb

1ESQ.pdb 2P58.pdb 3C3K.pdb 3I5W.pdb

1ESV.pdb 2PBX.pdb 3C4H.pdb 3I6O.pdb

1ET6.PDB 2PCA.pdb 3C4J.pdb 3I6Q.pdb

1ETA.pdb 2PER.pdb 3C4M.pdb 3I6V.pdb

1EUC-a.pdb 2PF0.pdb 3C58.pdb 3I77.pdb

1EUC-b.pdb 2PFB.pdb 3C5I.pdb 3I7S.pdb

1EUC.PDB 2PFC.pdb 3C63.pdb 3I8R.pdb

1EVX.PDB 2PGD.PDB 3C65.pdb 3I8W.pdb

1EWF.PDB 2PHK.pdb 3C68.pdb 3I9P.pdb

1EWX.pdb 2PJC.pdb 3C71.pdb 3I9S.pdb

1EWZ.PDB 2PK3.pdb 3C7J.pdb 3I9U.pdb

1EXR.PDB 2PKP.pdb 3C7M.pdb 3IA1.pdb

1EYE.PDB 2PMP.pdb 3C86.pdb 3IAL.pdb

1EZM.PDB 2PPC.pdb 3C8E.pdb 3IB8.pdb

1F09.PDB 2PQG.pdb 3C8I.pdb 3IBH.pdb

1F2U.pdb 2PR0.pdb 3C95.pdb 3IBW.pdb

1F37.PDB 2PRB.pdb 3C9H.pdb 3IC1.pdb

1F3U-a.pdb 2PRL.pdb 3C9P.pdb 3IC4.pdb

1F3U-a.txt 2PTV.pdb 3C9X.pdb 3ICD.PDB

1F3U-b.pdb 2PU3.pdb 3CA8.pdb 3ID4.pdb

1F3U-b.txt 2PVQ.pdb 3CAK.pdb 3IDA.pdb

1F3U.PDB 2PY0.pdb 3CB2.pdb 3IDF.pdb

1F47.pdb 2PYW.pdb 3CBC.pdb 3IE3.pdb

1f4l.pdb 2PYY.pdb 3CBU.pdb 3IEV.pdb

1f5m.pdb 2PZH.pdb 3CCD.pdb 3IG2.pdb

1F5Z.PDB 2Q0H.pdb 3CCK.pdb 3IGC.pdb

1FCA.PDB 2Q0J.pdb 3CCY.pdb 3IGR.pdb

1FDN.pdb 2Q1Y.pdb 3CDA.pdb 3IH4.pdb

1FDP.PDB 2Q2A.pdb 3CDE.pdb 3IHQ.pdb

1FEC.PDB 2Q3Z.pdb 3CDL.pdb 3IHS.pdb

1FFV-a.pdb 2Q5T.pdb 3CDX.pdb 3IHU.pdb

1FFV-b.pdb 2Q63.pdb 3CFW.pdb 3II1.pdb

1FFV-c.pdb 2Q6O.pdb 3CFZ.pdb 3IIE.pdb

1FFV.PDB 2Q6V.pdb 3CG3.pdb 3IIQ.pdb

1FH2.pdb 2Q89.pdb 3CGI.pdb 3IJD.pdb

1FIE.PDB 2Q8G.pdb 3CGZ.pdb 3IJH.pdb

1FIV.PDB 2Q96.pdb 3CH8.pdb 3IJT.pdb

1FJL.pdb 2Q9T.pdb 3CHY.PDB 3IJW.pdb

1FJL_DNA.PDB 2QAK.pdb 3CIH.pdb 3IKR.pdb

1FJM.PDB 2QB4.pdb 3CIJ.pdb 3IKT.pdb

1FKN.pdb 2QBU.pdb 3CIM.pdb 3IL7.pdb

1fl7-a.pdb 2QBW.pdb 3CJ5.pdb 3IL9.pdb

1fl7-b.pdb 2QCO.pdb 3CJN.pdb 3ILW.pdb

1fl7.pdb 2QCX.pdb 3CJY.pdb 3ILX.pdb

1FLP.PDB 2QDF.pdb 3CK1.pdb 3IMW.pdb

1FMC.PDB 2QDH.pdb 3CKM.pdb 3IOS.pdb

1FMW.pdb 2QEI.pdb 3CL0.pdb 3IP4.pdb

1FNA.PDB 2QEL.pdb 3CLA.PDB 3IPJ.pdb

1FNA.txt 2QFF.pdb 3CLK.pdb 3IPT.pdb

1FNB.PDB 2QFK.pdb 3CLO.pdb 3IQ5.pdb

1FNF_1.PDB 2QFN.pdb 3CMI.pdb 3IR3.pdb

1FP3.PDB 2QFP.pdb 3CMN.pdb 3IR4.pdb

1FSA.PDB 2QGH.pdb 3CMZ.pdb 3IRA.pdb

1FUS.PDB 2QGU.pdb 3CNM.pdb 3IRB.pdb

1FW3.pdb 2QIB.pdb 3CNR.pdb 3ISR.pdb

1FX5.PDB 2QJ7.pdb 3CNV.pdb 3ISZ.pdb

1fye.pdb 2QKA.pdb 3CO4.pdb 3IT7.pdb

1G16.PDB 2QLW.pdb 3CO8.pdb 3ITB.pdb

1G21-a.pdb 2QLX.pdb 3COL.pdb 3ITC.pdb

1G21-b.pdb 2QM8.pdb 3COO.pdb 3IUH.pdb

1G21-e.pdb 2QN3.pdb 3COP.pdb 3IUK.pdb

1G21.pdb 2QO0.pdb 3CP0.pdb 3IUU.pdb

1g6n.pdb 2QOP.pdb 3CP7.pdb 3IV4.pdb

1GAI.PDB 2QP2.pdb 3CPG.pdb 3IVE.pdb

1GCB.PDB 2QPJ.pdb 3CPO.pdb 3IWD.pdb

1GDJ.PDB 2QPQ.pdb 3CQJ.pdb 3IWG.pdb

1GDO.PDB 2QPZ.pdb 3CRA.pdb 3IWZ.pdb

1GER.PDB 2QQB.pdb 3CRT.pdb 3IX9.pdb

1GG1.PDB 2QRH.pdb 3CRY.pdb 3IXF.pdb

1GG4.PDB 2QRT.pdb 3CRZ.pdb 3IXJ.pdb

1GIF.PDB 2QRY.pdb 3CS2.pdb 3IXQ.pdb

1GND.PDB 2QS1.pdb 3CS5.pdb 3IXR.pdb

1GOF.PDB 2QSQ.pdb 3CSG.pdb 3JR1.pdb

1GOT-a.pdb 2QSX.pdb 3CSJ.pdb 3JR3.pdb

1GOT-b.pdb 2QT3.pdb 3CSX.pdb 3JR5.pdb

1GOT-g.pdb 2QTI.pdb 3CT2.pdb 3JRN.pdb

1GOT.PDB 2QTP.pdb 3CT5.pdb 3JSJ.pdb

1GOU.pdb 2QTT.pdb 3CT6.pdb 3JSN.pdb

1GOX.PDB 2QTZ.pdb 3CTA.pdb 3JSY.pdb

1GPL.PDB 2QU7.pdb 3CTG.pdb 3JTH.pdb

1GRN.PDB 2QU8.pdb 3CTP.pdb 3JTP.pdb

1GSP.PDB 2QUO.pdb 3CUO.pdb 3JU0.pdb

1GST.PDB 2QV3.pdb 3CUV.pdb 3JU4.pdb

1GST.txt 2QV7.pdb 3CV3.pdb 3JUD.pdb

1GTR.pdb 2QVP.pdb 3CV9.pdb 3JUK.pdb

1GUA.PDB 2QVV.pdb 3CWR.pdb 3JUQ.pdb

1GZM.pdb 2QWT.pdb 3CWX.pdb 3JVD.pdb

1H1W.pdb 2QWU.pdb 3CX4.pdb 3JW2.pdb

1H3E.pdb 2QWZ.pdb 3CXF.pdb 3JW4.pdb

1H4Q.pdb 2QXF.pdb 3CXG.pdb 3JWI.pdb

1H68.pdb 2QY2.pdb 3CXQ.pdb 3JY6.pdb

1H97.PDB 2QYQ.pdb 3CYE.pdb 3JYB.pdb

1HBG.PDB 2QZ8.pdb 3CYM.pdb 3JZ4.pdb

1HCK.PDB 2QZB.pdb 3CYN.pdb 3JZ9.pdb

1HCQ-старый.PDB 2QZC.pdb 3CZ4.pdb 3JZI.pdb

1HCQ.pdb 2QZK.pdb 3CZ8.pdb 3JZZ.pdb

1HCQ_1-старый.PDB 2QZW.pdb 3CZC.pdb 3K02.pdb

1HCR.PDB 2R19.pdb 3CZX.pdb 3K0L.pdb

1HCV.PDB 2R1I.pdb 3CZY.pdb 3K0P.pdb

1HDH.pdb 2R1P.pdb 3D02.pdb 3K1W.pdb

1HDS-a.pdb 2R2A.pdb 3D0O.pdb 3K29.pdb

1HDS-b.pdb 2R2H.pdb 3D0S.pdb 3K2C.pdb

1HDS.PDB 2R2U.pdb 3D1K.pdb 3K2M.pdb

1hgu.pdb 2R37.pdb 3D1R.pdb 3K3F.pdb

1HI1.pdb 2R38.pdb 3D1T.pdb 3K4F.pdb

1HP1.pdb 2R3X.pdb 3D1X.pdb 3K4V.pdb

1HPI.PDB 2R48.pdb 3D21.pdb 3K50.pdb

1HPM.PDB 2R4F.pdb 3D27.pdb 3K59.pdb

1HQ3-a.pdb 2R4Q.pdb 3D2M.pdb 3K5G.pdb

1HQ3-b.pdb 2R4T.pdb 3D2O.pdb 3K5O.pdb

1HQ3-c.pdb 2R50.pdb 3D2Y.pdb 3K5P.pdb

1HQ3-d.pdb 2R5F.pdb 3D30.pdb 3K5X.pdb

1HQ3.pdb 2R5G.pdb 3D3H.pdb 3K67.pdb

1HUR.PDB 2R5N.pdb 3D3I.pdb 3K69.pdb

1hwg.pdb 2R5O.pdb 3D3S.pdb 3K6X.pdb

1hwh.pdb 2R5W.pdb 3D43.pdb 3K83.pdb

1HYH.PDB 2R6O.pdb 3D4B.pdb 3K89.pdb

1HYP.PDB 2R6R.pdb 3D4F.pdb 3K8L.pdb

1I0E.pdb 2R75.pdb 3D4M.pdb 3K8N.pdb

1I1B.PDB 2R77.pdb 3D4P.pdb 3K8U.pdb

1I5Z.pdb 2R7A.pdb 3D55.pdb 3K9C.pdb

1IAG.PDB 2R7H.pdb 3D5J.pdb 3K9H.pdb

1IEA-a.pdb 2R8N.pdb 3D5L.pdb 3K9U.pdb

1IEA-b.pdb 2R8P.pdb 3D5R.pdb 3K9Z.pdb

1IEA.PDB 2R8T.pdb 3D5S.pdb 3KBK.pdb

1IFB.PDB 2R97.pdb 3D6I.pdb 3KBR.pdb

1IGD.PDB 2R99.pdb 3D79.pdb 3KCC.pdb

1IGN.PDB 2R9V.pdb 3D8P.pdb 3KCH.pdb

1IGN_A.PDB 2RA8.pdb 3DAQ.pdb 3KCO.pdb

1IGS.PDB 2RA9.pdb 3DB7.pdb 3KCP.pdb

1ILR.PDB 2RAE.pdb 3DBK.pdb 3KCZ.pdb

1INW.PDB 2RAM.PDB 3DBZ.pdb 3KD4.pdb

1IOW.PDB 2RB8.pdb 3DCJ.pdb 3KDC.pdb

1ISU.PDB 2RB9.pdb 3DDD.pdb 3KEB.pdb

1ITG.pdb 2RBD.pdb 3DDE.pdb 3KFM.pdb

1ITH.pdb 2RBH.pdb 3DDW.pdb 3KGC.pdb

1J09.pdb 2RCA.pdb 3DE9.pdb 3KGD.pdb

1J1D-a.pdb 2RCB.pdb 3DEU.pdb 3KGK.pdb

1J1D-b.pdb 2RCI.pdb 3DEW.pdb 3KGT.pdb

1J1D-c.pdb 2RD1.pdb 3DF8.pdb 3KH5.pdb

1J1D.pdb 2RD6.pdb 3DF9.pdb 3KH9.pdb

1J1V.pdb 2RDC.pdb 3DFG.pdb 3KHP.pdb

1j3h.pdb 2RDE.pdb 3DFT.pdb 3KJD.pdb

1J7K.pdb 2RDL.pdb 3DGB.pdb 3KJM.pdb

1JDD.PDB 2RDP.pdb 3DGD.pdb 3KJT.pdb

1JER.PDB 2RDQ.pdb 3DGT.pdb 3KJX.pdb

1JI0.pdb 2RDS.pdb 3DH2.pdb 3KJY.pdb

1JJV.pdb 2REE.pdb 3DH8.pdb 3KKE.pdb

1JMC.PDB 2REG.pdb 3DHC.pdb 3KKW.pdb

1JOI.PDB 2REK.pdb 3DHP.pdb 3KLR.pdb

1K4C-a.pdb 2REM.pdb 3DHV.pdb 3KM4.pdb

1K4C-b.pdb 2RF1.pdb 3DID.pdb 3KMI.pdb

1K4C-c.pdb 2RFG.pdb 3DIE.pdb 3KMN.pdb

1K4C.pdb 2RFQ.pdb 3DK5.pdb 3KOJ.pdb

1K8K-a.pdb 2RG7.pdb 3DKD.pdb 3KOT.pdb

1K8K-b.pdb 2RGH.pdb 3DKS.pdb 3KP7.pdb

1K8K-c.pdb 2RGS.pdb 3DL2.pdb 3KP8.pdb

1K8K-d.pdb 2RGV.pdb 3DLO.pdb 3KPT.pdb

1K8K-e.pdb 2RGY.pdb 3DML.pdb 3KQJ.pdb

1K8K-f.pdb 2RHG.pdb 3DNC.pdb 3KR6.pdb

1K8K-g.pdb 2RHJ.pdb 3DNJ.pdb 3KRE.pdb

1K8K.pdb 2RID.pdb 3DNU.pdb 3KS9.pdb

1KBU.pdb 2RIN.pdb 3DNX.pdb 3KSM.pdb

1KCM.pdb 2RJO.pdb 3DO8.pdb 3KT0.pdb

1KEV.PDB 2RJZ.pdb 3DOR.pdb 3KTC.pdb

1kf9-a.pdb 2RK2.pdb 3DP4.pdb 3KTL.pdb

1kf9-b.pdb 2RKQ.pdb 3DP6.pdb 3KTW.pdb

1kf9.pdb 2RL1.pdb 3DP9.pdb 3KUO.pdb

1KFG.PDB 2RN2.PDB 3DQY.pdb 3KV1.pdb

1KIT.PDB 2RSP.PDB 3DR0.pdb 3KVM.pdb

1KJ8.pdb 2SN3.PDB 3DR6.pdb 3KVO.pdb

1KK9.pdb 2ST1.PDB 3DR7.pdb 3KW3.pdb

1KO5.pdb 2STV.PDB 3DR8.pdb 3KWE.pdb

1KO9.pdb 2TMY.pdb 3DR9.pdb 3KWO.pdb

1KON.pdb 2UVH.pdb 3DRE.pdb 3KWP.pdb

1KOQ.pdb 2UVK.pdb 3DRN.pdb 3KX6.pdb

1KP2.pdb 2UZ2.pdb 3DSB.pdb 3KX7.pdb

1KP3.pdb 2UZJ.pdb 3DSG.pdb 3KYF.pdb

1KP4.pdb 2UZP.pdb 3DSQ.pdb 3KYG.pdb

1KP8-A.pdb 2V00.pdb 3DSZ.pdb 3KYJ.pdb

1KP8.pdb 2V04.pdb 3DUE.pdb 3KZ9.pdb

1KQ1.pdb 2V0C.pdb 3DUW.pdb 3KZG.pdb

1KQ6.pdb 2V0L.pdb 3DV2.pdb 3KZL.pdb

1KQP.pdb 2V1K.pdb 3DV5.pdb 3KZP.pdb

1KR1.pdb 2V1M.pdb 3DVW.pdb 3L0H.pdb

1KR2-A.pdb 2V25.pdb 3DWC.pdb 3L1M.pdb

1KR2.pdb 2V2J.pdb 3DWF.pdb 3L1V.pdb

1KRH.pdb 2V32.pdb 3DWV.pdb 3L31.pdb

1KSL.pdb 2V3Q.pdb 3DX5.pdb 3L3N.pdb

1KVK.pdb 2V3V.pdb 3DXI.pdb 3L4N.pdb

1KX5-a.pdb 2V4C.pdb 3DXP.pdb 3L5F.pdb

1KX5-b.pdb 2V4M.pdb 3DYR.pdb 3L5I.pdb

1KX5-c.pdb 2V4N.pdb 3DZC.pdb 3L68.pdb

1KX5-d.pdb 2V58.pdb 3E0H.pdb 3L6G.pdb

1KX5.pdb 2V5I.pdb 3E0U.pdb 3L6I.pdb

1L2T.pdb 2V65.pdb 3E11.pdb 3L6U.pdb

1L3A.pdb 2V6G.pdb 3E1S.pdb 3LAT.pdb

1L8J.pdb 2V6K.pdb 3E27.pdb 3LC0.pdb

1LAM.PDB 2V6O.pdb 3E2C.pdb 3LCE.pdb

1LAT.PDB 2V73.pdb 3E3E.pdb 3LD7.pdb

1LCF.PDB 2V77.pdb 3E3M.pdb 3LEZ.pdb

1LCI.PDB 2V7P.pdb 3E3U.pdb 3LFT.pdb

1LCU.pdb 2V7X.pdb 3E3V.pdb 3LFZ.pdb

1LHR.pdb 2V84.pdb 3E3X.pdb 3LG6.pdb

1lhs.pdb 2V8U.pdb 3E4F.pdb 3LGA.pdb

1LIT.PDB 2V9E.pdb 3E4R.pdb 3LGE.pdb

1LMB.PDB 2V9V.pdb 3E4W.pdb 3LGK.pdb

1LML.PDB 2VAP.pdb 3E51.pdb 3LGS.pdb

1LN3.pdb 2VBA.pdb 3E5N.pdb 3LHE.pdb

1LPE.PDB 2VBQ.pdb 3E5X.pdb 3LHF.pdb

1LQ1.pdb 2VBU.pdb 3E61.pdb 3LHG.pdb

1LQV.pdb 2VBW.pdb 3E6B.pdb 3LHK.pdb

1LST.PDB 2VCC.pdb 3E6D.pdb 3LHN.pdb

1LTS-a.pdb 2VCO.pdb 3E7B.pdb 3LHQ.pdb

1LTS-c.pdb 2VD4.pdb 3E7L.pdb 3LJ7.pdb

1LTS-d.pdb 2VD9.pdb 3E7Q.pdb 3LKL.pdb

1LTS.PDB 2VDZ.pdb 3E8P.pdb 3LKV.pdb

1LUC.PDB 2VES.pdb 3E9I.pdb 3LLO.pdb

1LZ1.PDB 2VFQ.pdb 3EAF.pdb 3LLX.pdb

1MA7.pdb 2VG1.pdb 3EBP.pdb 3LM7.pdb

1MAU.pdb 2VG2.pdb 3EC0.pdb 3LMB.pdb

1MB9.pdb 2VG3.pdb 3EC0.txt 3LMF.pdb

1MBA.PDB 2VGK.pdb 3EC1.pdb 3LOP.pdb

1MDC.PDB 2VGQ.pdb 3EC3.pdb 3LOQ.pdb

1MDU-a.pdb 2VGX.pdb 3EC4.pdb 3LOR.pdb

1MDU-b.pdb 2VHA.pdb 3ECH.pdb 3LOU.pdb

1MDU.pdb 2VHD.pdb 3ED3.pdb 3LP1.pdb

1MEY.PDB 2VID.pdb 3EDO.pdb 3LPK.pdb

1MIW.pdb 2VIM.pdb 3EDP.pdb 3LQ3.pdb

1MJH.pdb 2VK2.pdb 3EDS.pdb 3LQ4.pdb

1MJM.pdb 2VKE.pdb 3EIN.pdb 3LQ7.pdb

1MNM-a.pdb 2VKG.pdb 3EIR.pdb 3LQF.pdb

1MNM-c.pdb 2VKL.pdb 3EIV.pdb 3LQK.pdb

1MNM.PDB 2VKM.pdb 3EJW.pdb 3LQN.pdb

1MOL.PDB 2VKU.pdb 3EL5.pdb 3LQS.pdb

1MUP.PDB 2VKV.pdb 3ELF.pdb 3LR1.pdb

1MWC.PDB 2VKW.pdb 3ELG.pdb 3LSS.pdb

1MWD.pdb 2VL3.pdb 3ELK.pdb 3LSW.pdb

1MXD.pdb 2VLG.pdb 3ELQ.pdb 3LSZ.pdb

1myt.pdb 2VLQ.pdb 3EM6.pdb 3LTH.pdb

1MZM.PDB 2VLX.pdb 3EMF.pdb 3LUK.pdb

1N48.pdb 2VM2.pdb 3EMI.pdb 3LUR.pdb

1N4L-a.pdb 2VMB.pdb 3EMR.pdb 3LUU.pdb

1N4L.pdb 2VN5.pdb 3EMX.pdb 3LUY.pdb

1N84.pdb 2VN6.pdb 3EMY.pdb 3LV0.pdb

1NAH.PDB 2VND.pdb 3ENU.pdb 3LVM.pdb

1NCI.PDB 2VNK.pdb 3EO4.pdb 3LW9.pdb

1NFK.PDB 2VOB.pdb 3EO5.pdb 3LWA.pdb

1NFP.PDB 2VOC.pdb 3EO6.pdb 3LWC.pdb

1NLV-a.pdb 2VOZ.pdb 3EOI.pdb 3LWF.pdb

1NLV-g.pdb 2VPA.pdb 3EOJ.pdb 3LWG.pdb

1NLV.pdb 2VPO.pdb 3EP1.pdb 3LWJ.pdb

1NNC.PDB 2VPQ.pdb 3EPX.pdb 3LWU.pdb

1NOY.PDB 2VQ2.pdb 3ERG.pdb 3LXT.pdb

1NSF.pdb 2VQY.pdb 3ERV.pdb 3LXX.pdb

1NYR.pdb 2VR3.pdb 3ERX.pdb 3LXY.pdb

1OBD.pdb 2VRQ.pdb 3ESM.pdb 3LXZ.pdb

1OCR-a.pdb 2VRY.pdb 3ESP.pdb 3LYD.pdb

1OCR-b.pdb 2VSZ.pdb 3ESS.pdb 3LYH.pdb

1OCR-c.pdb 2VT1.pdb 3ETT.pdb 3LYP.pdb

1OCR-d.pdb 2VU1.pdb 3ETZ.pdb 3LYQ.pdb

1OCR-e.pdb 2VU5.pdb 3EU4.pdb 3LYY.pdb

1OCR-f.pdb 2VUC.pdb 3EU8.pdb 3LZ7.pdb

1OCR-g.pdb 2VUP.pdb 3EUP.pdb 3LZK.pdb

1OCR-h.pdb 2VUZ.pdb 3EUR.pdb 3LZM.PDB

1OCR-i.pdb 2VVS.pdb 3EVO.pdb 3M0Z.pdb

1OCR-j.pdb 2VVT.pdb 3EVY.pdb 3M10.pdb

1OCR-k.pdb 2VW2.pdb 3EWA.pdb 3M1M.pdb

1OCR-L.pdb 2VX9.pdb 3EWG.pdb 3M2T.pdb

1OCR-m.pdb 2VXK.pdb 3EWL.pdb 3M34.pdb

1OCR.PDB 2VXY.pdb 3EX9.pdb 3M3B.pdb

1OEW.pdb 2VY0.pdb 3EXN.pdb 3M3M.pdb

1OIS.pdb 2VY9.pdb 3EY5.pdb 3M4R.pdb

1OKC.pdb 2VYW.pdb 3EYE.pdb 3M5K.pdb

1oks.pdb 2VZB.pdb 3EYT.pdb 3M5V.pdb

1omw-a.pdb 2VZR.pdb 3F0I.pdb 3M5Z.pdb

1omw-b.pdb 2W02.pdb 3F10.pdb 3M6L.pdb

1omw-g.pdb 2W0V.pdb 3F11.pdb 3M6Y.pdb

1omw.pdb 2W19.pdb 3F1T.pdb 3M70.pdb

1OS1.pdb 2W1K.pdb 3F2H.pdb 3M7I.pdb

1OTG.PDB 2W1V.pdb 3F2R.pdb 3M7V.pdb

1OVA.PDB 2W20.pdb 3F39.pdb 3M8N.pdb

1P6C.pdb 2W22.pdb 3F3Q.pdb 3M9F.pdb

1P78.pdb 2W2B.pdb 3F3X.pdb 3M9V.pdb

1PAM.PDB 2W38.pdb 3F43.pdb 3MA4.pdb

1PDA.PDB 2W3J.pdb 3F4J.pdb 3MAH.pdb

1PDN.pdb 2W3Z.pdb 3F4T.pdb 3MB5.pdb

1PGB.PDB 2W46.pdb 3F64.pdb 3MBD.pdb

1PHH.PDB 2W47.pdb 3F6D.pdb 3MBP.pdb

1PHK.pdb 2W53.pdb 3F6F.pdb 3MC4.pdb

1PHP.PDB 2W7S.pdb 3F6J.pdb 3MC9.pdb

1PII.PDB 2W7Y.pdb 3F6O.pdb 3MDK.pdb

1PLC.PDB 2W87.pdb 3F6V.pdb 3ME8.pdb

1PLQ.PDB 2W8W.pdb 3F7C.pdb 3MEP.pdb

1POX.PDB 2W9T.pdb 3F7D.pdb 3MEQ.pdb

1PSG.PDB 2WA8.pdb 3F85.pdb 3MES.pdb

1PTA.PDB 2WAN.pdb 3F8F.pdb 3MFB.pdb

1PVP.pdb 2WB9.pdb 3F8K.pdb 3MGD.pdb

1PVU.PDB 2WBN.pdb 3F8M.pdb 3MGL.pdb

1Q24.pdb 2WC1.pdb 3F9Q.pdb 3MHG.pdb

1Q97.pdb 2WCI.pdb 3F9U.pdb 3MI2.pdb

1QBB.PDB 2WCX.pdb 3FAS.pdb 3MIX.pdb

1QHB-a.pdb 2WCZ.pdb 3FB3.pdb 3MIZ.pdb

1QHB.PDB 2WDA.pdb 3FB9.pdb 3MKH.pdb

1QHG.pdb 2WDF.pdb 3FBU.pdb 3MKL.pdb

1QHH-a.pdb 2WDU.pdb 3FCE.pdb 3MN2.pdb

1QHH-b.pdb 2WDZ.pdb 3FCH.pdb 3MOG.pdb

1QHH-c.pdb 2WE8.pdb 3FD6.pdb 3MPD.pdb

1QHH-d.pdb 2WFC.pdb 3FDX.pdb 3MQZ.pdb

1QHH.pdb 2WFG.pdb 3FEG.pdb 3MSZ.pdb

1QHT.PDB 2WFJ.pdb 3FF9.pdb 3MT0.pdb

1QLP.pdb 2WFW.pdb 3FFE.pdb 3MT1.pdb

1QME.PDB 2WHN.pdb 3FFO.pdb 3MTJ.pdb

1QNH.pdb 2WHO.pdb 3FFV.pdb 3MTQ.pdb

1R3E.pdb 2WIU.pdb 3FFY.pdb 3MTR.pdb

1R8B.pdb 2WJ1.pdb 3FGC.pdb 3MTX.pdb

1RCD.PDB 2WJR.pdb 3FGR.pdb 3MU9.pdb

1RCF.PDB 2WJW.pdb 3FH0.pdb 3MUQ.pdb

1REQ-a.pdb 2WK0.pdb 3FH4.pdb 3MUX.pdb

1REQ-b.pdb 2WK1.pdb 3FH9.pdb 3MVC.pdb

1REQ.PDB 2WKE.pdb 3FHD.pdb 3MVP.pdb

1RGE.PDB 2WKW.pdb 3FHU.pdb 3MVU.pdb

1RH0-A.pdb 2WKX.pdb 3FHV.pdb 3MWH.pdb

1RH0.pdb 2WKY.pdb 3FI6.pdb 3MX0.pdb

1rl3.pdb 2WL0.pdb 3FI7.pdb 3MXU.pdb

1RNL.PDB 2WL1.pdb 3FI8.pdb 3MY7.pdb

1ROP.PDB 2WLD.pdb 3FID.pdb 3MZ1.pdb

1RRJ.pdb 2WLG.pdb 3FIU.pdb 3MZN.pdb

1RRP.PDB 2WLT.pdb 3FIW.pdb 3N0S.pdb

1RVA.PDB 2WLW.pdb 3FIX.pdb 3N0V.pdb

1RXW.pdb 2WNW.pdb 3FJ1.pdb 3N0W.pdb

1RYB.pdb 2WO4.pdb 3FJ4.pdb 3N10.pdb

1RYT.PDB 2WOD.pdb 3FJM.pdb 3N26.pdb

1RZL.pdb 2WOE-a.pdb 3FJU.pdb 3N29.pdb

1S9J.pdb 2WOE.pdb 3FK5.pdb 3N2B.pdb

1SAR.PDB 2WOQ.pdb 3FK6.pdb 3N2O.pdb

1SBP.PDB 2WPT.pdb 3FK7.pdb 3N3S.pdb

1SCT-a.pdb 2WPX.pdb 3FK8.pdb 3N3W.pdb

1SCT-b.pdb 2WQ1.pdb 3FKF.pdb 3N4F.pdb

1SCT.PDB 2WQF.pdb 3FKJ.pdb 3N55.pdb

1SE4.PDB 2WQX.pdb 3FKT.pdb 3N5L.pdb

1SET.PDB 2WR8.pdb 3FM5.pdb 3N5O.pdb

1SK6-a.PDB 2WR9.pdb 3FMC.pdb 3N73.pdb

1sk6-d.pdb 2WRA.pdb 3FMS.pdb 3NAQ.pdb

1sk6.pdb 2WRZ.pdb 3FN7.pdb 3NDJ.pdb

1SMD.pdb 2WSB.pdb 3FND.pdb 3NFU.pdb

1SPB_a.PDB 2WSJ.pdb 3FNK.pdb 3NG3.pdb

1SPB_b.pdb 2WTG.pdb 3FPN.pdb 3PEP.pdb

1SRR.PDB 2WU9.pdb 3FPW.pdb 3PGK.pdb

1SU2.pdb 2WUA.pdb 3FQ6.pdb 3PJR.pdb

1SYX-A.PDB 2WUK.pdb 3FRC.pdb 3PMG.PDB

1SYX-b.pdb 2WV1.pdb 3FRQ.pdb 3PVI.PDB

1SYX.pdb 2WW8.pdb 3FSI.pdb 3RAB.PDB

1T27.pdb 2WWD.pdb 3FSM.pdb 3SDH.PDB

1TAD.PDB 2WWX.pdb 3FTN.pdb 3SLI.pdb

1TAQ.PDB 2WY4.pdb 3FU1.pdb 3SSI.pdb

1tb7.pdb 2WYH.pdb 3FV7.pdb 3WRP.PDB

1TEN.PDB 2WZ9.pdb 3FVO.pdb 3XIS.pdb

1TF4.PDB 2X0K.pdb 3FW2.pdb 451C.pdb

1TFK.pdb 2X26.pdb 3FW6.pdb 4CRX.PDB

1THB-a.pdb 2X3H.pdb 3FWE.pdb 4DMR.pdb

1THB-b.pdb 2X5Y.pdb 3FWJ.pdb 4ENL.PDB

1THB.PDB 2X76.pdb 3FWM.pdb 4FIV.pdb

1THT.PDB 2X7K.pdb 3FX3.pdb 4ICB.PDB

1TIB.PDB 2X7K.txt 3FXU.pdb 4MBP.pdb

1TID.pdb 2X8L.pdb 3FYB.pdb 4PEP.pdb

1TII-a.PDB 2X97.pdb 3FYD.pdb 4SLI.pdb

1TII-c.pdb 2X98.pdb 3FYH.pdb 4TMS.PDB

1TII-d.pdb 2X9G.pdb 3FYN.pdb 4TS1.PDB

1TII.PDB 2X9V.pdb 3FYZ.pdb 4TV8-a.pdb

1TML.pdb 2XCC.pdb 3FZ4.pdb 4TV8-b.pdb

1TQP.pdb 2XCY.pdb 3FZW.pdb 4TV8-f.pdb

1TRO-a.pdb 2XD3.pdb 3G06.pdb 4TV8.pdb

1TRO.pdb 2XDH.pdb 3G15.pdb 5ACN.PDB

1TRY.PDB 2XDN.pdb 3G1O.pdb 5CHY.pdb

1TSR.PDB 2XIS.pdb 3G1W.pdb 5CNA.PDB

1TUP.pdb 2YR2.pdb 3G1Z.pdb 5NUL.PDB

1TUP_A.PDB 2YSK.pdb 3G20.pdb 5TIM.PDB

1u7e-a.pdb 2YV7.pdb 3G23.pdb 5TNC.PDB

1u7e-b.pdb 2YV9.pdb 3G27.pdb 6CEL.pdb

1u7e.pdb 2YVE.pdb 3G2B.pdb 6GSP.pdb

1UBQ.PDB 2YVJ.pdb 3G2M.pdb 6LDH.pdb

1UCW.PDB 2YVT.pdb 3G2N.pdb 6Q21.PDB

1UDR.PDB 2YVW.pdb 3G3H.pdb 6XIA.pdb

1UEV.pdb 2YWH.pdb 3G3O.pdb 7CEL.pdb

1UIP.PDB 2YWI.pdb 3G3P.pdb 7NN9.pdb

1UTG.PDB 2YWM.pdb 3G3U.pdb 7RSA.PDB

1UVM.pdb 2YWN.pdb 3G3Z.pdb 7TIM.PDB

1VC9.pdb 2YWO.pdb 3G4P.pdb 7XIA.PDB

1VCA.PDB 2YWV.pdb 3G56.pdb 8ACN.pdb

1VCC.PDB 2YX4.pdb 3G5I.pdb 8CAT.PDB

1VCI.pdb 2YX7.pdb 3G67.pdb 8ICN.pdb

1VDN.pdb 2YXB.pdb 3G68.pdb 9ABP.pdb

1VIN.PDB 2YXX.pdb 3G69.pdb 9LDT.PDB

1VJD.pdb 2YXY.pdb 3G72.pdb 9PAP.PDB

1VLB.pdb 2YYE.pdb 3G77.pdb 9RNT.pdb

1VPT.PDB 2YZH.pdb 3G7J.pdb list.bat

1VRT-a.pdb 2YZK.pdb 3G7R.pdb list.xlsm

1VRT-b.pdb 2YZL.pdb 3G7W.pdb

1VRT.PDB 2YZU.pdb 3G7Z.pdb
